# Construction and validation of a novel SUMOylation-related lncRNAs signature for predicting the prognosis, tumor immune microenvironment, and therapeutic sensitivity of lung adenocarcinoma

**DOI:** 10.1016/j.gendis.2024.101338

**Published:** 2024-05-28

**Authors:** Jie Cai, Weizhong Ruan, Zida Wang, Gongzhe Liu, Bei Yang, Hao Wang, Deping Zhao, Chang Chen, Xiaogang Zhao

**Affiliations:** a Department of Thoracic Surgery, Shanghai Pulmonary Hospital, School of Medicine, Tongji University, Shanghai 200433, China; b Department of Thoracic Surgery, Clinical Oncology School of Fujian Medical University, Fujian Cancer Hospital (Fujian Branch of Fudan University Shanghai Cancer Center), Fuzhou, Fujian 350014, China; c Department of Emergency, Shanghai Pulmonary Hospital, School of Medicine, Tongji University, Shanghai 200433, China; d Department of Cardiothoracic Surgery, People's Hospital Affiliated to Shandong First Medical University, Jinan, Shandong 271199, China

Lung adenocarcinoma (LUAD) is the most prevalent subtype of lung cancer and the leading cause of cancer deaths worldwide.^1^ Despite immunotherapy, radiotherapy, and chemotherapy advances, treatment outcomes remain unsatisfactory.^2^ Thus, prognostic models that accurately predict patient prognosis and guide individualized treatment are desperately needed. SUMOylation, a reversible post-translational modification, is a crucial molecular regulatory mechanism that affects tumor progression.^3^ However, the role of sumoylation-related lncRNAs (SR-lncRNAs) in LUAD has not been explored. This study aimed to construct and validate an SR-lncRNAs signature for predicting LUAD prognosis, tumor immune microenvironment, and therapeutic sensitivity.

Figure S1 shows the study's flowchart. Firstly, after removing genes expressed in less than half of patients, the TCGA_LUAD dataset yielded 15,831 lncRNAs. Then, 2858 lncRNAs were identified as differentially expressed lncRNAs based on false discovery rate < 0.01 and |log_2_ fold change| ≥ 1.0 (Fig. S2). We found 187 sumoylation-related genes in the Molecular Signatures Database (https://www.gsea-msigdb.org/gsea/msigdb/index.jsp) and collected expression data from the TCGA_LUAD dataset. Pearson's correlation analyses between differentially expressed lncRNAs and sumoylation-related genes yielded 831 SR-lncRNAs. Univariate Cox regression analyses further identified 54 prognostic SR-lncRNAs with *p* values < 0.01. To create a reliable risk predictive model, patients were randomly and equally divided into training (*n* = 245) and testing (*n* = 245) cohorts. The aforementioned 54 SR-lncRNAs were analyzed using LASSO Cox regression and cross-validated, and 20 prognostic lncRNAs were selected (Fig. 1A, B). Step-by-step multivariate Cox regression analysis further purified 7 lncRNAs for improved clinical utility (Fig. S3). Finally, a prognostic risk model was developed utilizing the correlation coefficient of the expression levels of lncRNAs: risk score = (0.4295 × status of OGFRP1) + (0.3006 × status of PRKG1-AS1) + (0.2636 × status of AL353746.1) + (0.1115 × status of SATB2-AS1) + (−0.0850 × status of FTO-IT1) + (−0.3837 × status of MED4-AS1) + (−0.4211 × status of AC090559.1).

**Figure 1:**
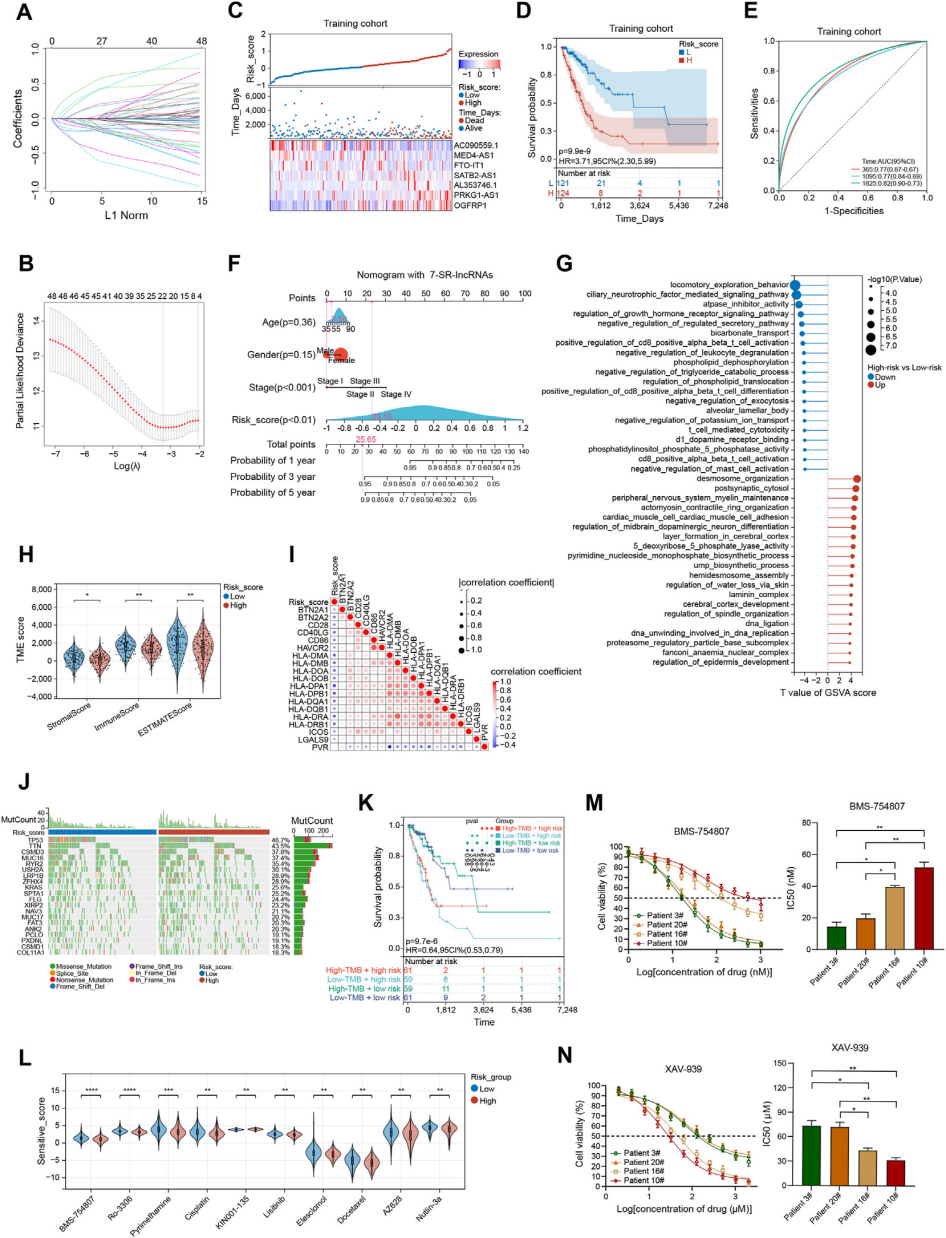
A novel SUMOylation-related lncRNA signature for predicting the prognosis, tumor immune microenvironment, and therapeutic sensitivity of lung adenocarcinoma. **(A)** Profiles of LASSO coefficients for 54 prognostic SR-lncRNAs. **(B)** Ten-fold cross-validation for selecting the LASSO Cox regression model's tuning parameter lambda. The red dots represent the partial likelihood of deviance values, the grey lines represent standard error (SE), and the two vertical dashed lines on the left and right represent optimal values by minimal and 1-SE criteria, respectively. **(C)** The distribution of risk scores, OS status, and expression profiles of the 7 SR-lncRNAs in the training cohort. **(D)** Kaplan–Meier curve for OS in the training cohort. **(E)** The receiver operating characteristic (ROC) curves show the potential of the 7-SR-lncRNAs signature in predicting 1-, 3-, and 5-year OS in the training cohort. **(F)** Nomograms integrated with the 7-SR-lncRNAs signature for predicting OS probability in the training cohort. **(G)** Gene Set Variation Analysis (GSVA) between low- and high-risk groups. **(H)** Comparison of the stromal score, immune score, and ESTIMATE score between low- and high-risk groups. **(I)** The correlation heatmap displays the associations between risk score and 19 differentially expressed immune checkpoint genes. **(J)** The waterfall plot of somatic mutation features in two groups. **(K)** Kaplan–Meier analysis of OS for patients classified by combing tumor mutation burden (TMB) status and risk score. **(L)** The top 10 differentially sensitive drugs between low- and high-risk groups. **(M)** Cytotoxicity assays of BMS-754807 in patient-derived cells (PDCs) constructed from high- (patients 3# and 20#) and low-risk patients (patients 10# and 16#). **(N)** Cytotoxicity assays of XAV-939 in PDCs constructed from high- (patients 3# and 20#) and low-risk patients (patients 10# and 16#). SR-lncRNAs, sumoylation-related lncRNAs; OS, overall survival.

LUAD patients in the training cohort were categorized as low- or high-risk by median risk score (0.058140853). The survival distribution curve showed a greater death rate for high-risk patients. Heatmap showed that, in the high-risk group, 4 hazardous lncRNAs were highly expressed, while the others were down-regulated (Fig. 1C). The Kaplan–Meier plot demonstrated a lower overall survival rate for the high-risk group (Fig. 1D). Good performance was shown by receiver operating characteristic (ROC) curves with values of 0.77 at 1 year, 0.77 at 3 years, and 0.82 at 5 years for area under the curve (Fig. 1E). Furthermore, compared with previously published prognostic models and clinicopathological risk factors (such as age, gender, and TNM stage), our signature had better prognostic prediction value (Fig. S4). Similar results were found in the testing cohort and the whole cohort (Fig. S5). To make the 7-SR-lncRNAs signature clinically useful, nomograms were created by combining them with or without clinicopathological risk factors (Fig. 1F; Fig. S6A). Calibration curves showed that the 7-SR-lncRNAs-integrated nomogram was more consistent with observation than the simple clinicopathologic nomogram (Fig. S6B, C). The 7-SR-lncRNAs-integrated nomogram may be superior for LUAD clinical application because the values for its area under the curve at 1, 3, and 5 years were significantly higher (Fig. S6D, E).

Furthermore, numerous approaches were used to investigate the biological activities of the 7-SR-lncRNAs risk model. The Gene Set Variation Analysis (GSVA) found that the two risk groups had differing enrichments of cancer-related pathways like regulation of growth hormone receptor signaling pathway, positive regulation of CD8^+^ alpha beta T-cell activation, and T-cell mediated cytotoxicity (Fig, 1G). In the Gene Set Enrichment Analysis (GSEA) plots, these SR-lncRNAs were enriched in sumoylation, metabolism, and immunology pathways (Fig. S7A–G). Co-expression of protein-coding genes with the 7 SR-lncRNAs was identified using a criterion of |Pearson correlation coefficient| > 0.5 and *p* < 0.001. Gene Ontology (GO) and Kyoto Encyclopedia of Genes and Genomes (KEGG) analyses revealed that those genes were enriched in immune response, Th1/Th2 cell differentiation, and B cell receptor signaling (Fig. S7H, I). In addition to its relevance to classical cancer-related pathways, these findings revealed that the 7-SR-lncRNAs signature was also linked to the immune response.

To study the risk model's role in the tumor immune microenvironment, immune cell infiltration in tumor samples was examined. CIBERSORT, EPIC, MCPCounter, quanTIseq, TIMER, and xCell identified distinct immune cell expression in low- and high-risk groups (Fig. S8A). Using the xCell algorithm, we observed that low-risk samples had more infiltrating immune cells, such as CD4^+^ T-cells, CD8^+^ T-cells, and monocytes (Fig. S8A). Additionally, ESTIMATE showed statistically significant stromal activity variation (Fig. 1H). In light of the development of immune checkpoint inhibitors, we further examined the expression of 79 immune checkpoint genes in low- and high-risk groups,^4^ and found 19 of them were differentially expressed (Fig. S8B). The correlation heatmap showed that these 19 immune checkpoint genes were strongly linked with the risk score (Fig. 1I). These data demonstrated that the 7-SR-lncRNAs signature might assess the tumor immune microenvironment and immune checkpoint gene expression in LUAD patients.

Tumor mutation burden (TMB) can predict immune checkpoint inhibitor responses.^5^ The TMB of low- and high-risk LUAD patients is shown in Fig. 1J. However, the difference in TMB levels between the two groups was not statistically significant (*P* = 0.09; Fig. S9A), and the groups with low- or high-TMB levels had similar survival rates (*P* = 0.47; Fig. S9B). Subgroup analysis stratified samples by TMB status and risk groups was further performed. Intriguingly, some patients with high-risk scores in the low- or high-TMB groups had significantly shorter overall survival than those with low-risk scores, but no significance was seen between TMB groups with the same risk ratings (Fig. 1K). These findings suggested that the combination of risk group and TMB could provide a more accurate estimate of patient prognosis.

The disparity between the tumor immune microenvironment and TMB may result in differential susceptibility to drug response. We used the “pRRophetic” R package to compare pharmacological efficacy across high- and low-risk groups and found that 45 drugs had statistically significant differences (*P* < 0.05; Fig. S10). Among the top 10 differentially sensitive drugs, BMS-754807, Ro-3306, pyrimethamine, cisplatin, elesclomol, docetaxel, AZ628, and nutlin-3a may be more effective in high-risk patients, and low-risk patients may benefit more from KIN001-135 and lisitinib (Fig. 1L).

A clinical cohort of 80 LUAD patients was created to externally confirm the stability of the 7-SR-lncRNAs signature. Quantitative reverse transcription PCR showed that LUAD tissues expressed more SATB2-AS1, AL353746.1, PRKG1-AS1, and OGFRP1 than neighboring normal tissues, but less AC090559.1, MED4-AS1, and FTO-IT1 (Fig. S11A). In the clinical cohort, the 7 SR-lncRNAs exhibited similar risk scores, overall survival status, and expression profiles to the training cohort (Fig. S11B). The Kaplan–Meier curve and ROC curve indicated that the model could effectively predict prognosis in the clinical cohort as well (Fig. S11C, D). Finally, based on the differing sensitivity profiles (Fig. S10), we validated the sensitivity difference of BMS-754807 and XAV-939 in patient-derived cells obtained from high- and low-risk LUAD clinical samples. The dose–response curves showed that BMS-754807 was more effective in high-risk patients (patients 3# and 20#; Fig. 1M), while low-risk patients (patients 10# and 16#) were more sensitive to XAV-939 (Fig. 1N).

To the best of our knowledge, this is the first study to develop an SR-lncRNAs predictive signature in patients with LUAD and validate it in an independent clinical cohort. This risk model showed good diagnostic accuracy in predicting patient survival outcomes and helped optimize individual treatment strategies. Patient stratification may be improved and novel drugs can be explored in distinct risk groups by making use of differences in features between low- and high-risk groups, such as immune infiltration, TMB, and drug sensitivity. We anticipate eagerly future research into the mechanisms by which these SR-lncRNAs regulate sumoylation and impact patient outcomes.

## Ethics declaration

The collection of human tissues was approved by the Medical Ethics Committee of Shanghai Pulmonary Hospital (Approval number: K23-075Y). Written informed consent was obtained from each patient.

## Author contributions

Conception and design: J.C., X.Z., C.C., and D.Z. Collection and assembly of data: J.C., Z.W., and G.L. Data analysis and interpretation: J.C., W.R., B.Y., and H.W. Conducting experiments: J.C., W.R., and Z.W. Manuscript writing and revisions: J.C., X.Z., C.C., and D.Z. Final approval of manuscript: all authors. Accountable for all aspects of work: all authors. All authors read and agreed to the published version of the manuscript.

## Conflict of interests

The authors have no conflict of interests to declare.

## Funding

This work was supported by the Shanghai Sailing Program (China) (No. 22YF1437600 to J.C.) and the Programs of Shanghai Pulmonary Hospital (China) (No. fkzr2477 to J.C.).

## Data availability

All data are available. Please contact us to access it if it is needed.

## Figures and Tables

**Figure S6:**
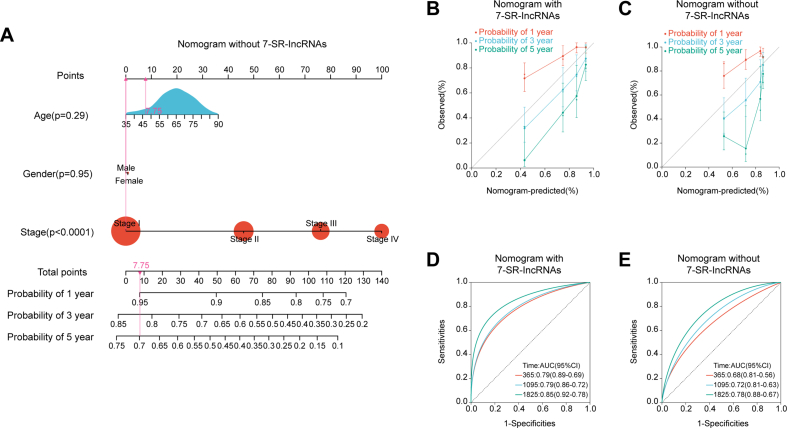
Construction of a nomogram based on the 7-SR-lncRNAs signature. **(A)** Nomograms integrated without the 7-SR-lncRNAs signature for predicting OS probability in the training cohort. **(B, C)** Calibration curves for the 7-SR-lncRNAs-integrated nomogram (B) and the simple clinicopathologic nomogram (C) indicate the calibration of each model in terms of the agreement between predicted and observed outcomes. **(D, E)** The receiver operating characteristic (ROC) curves show the potential of the 7-SR-lncRNAs-integrated nomogram (D) and the simple clinicopathologic nomogram (E) in predicting 1-, 3-, and 5-year OS. SR-lncRNAs, sumoylation-related lncRNAs; OS, overall survival.

**Figure S7:**
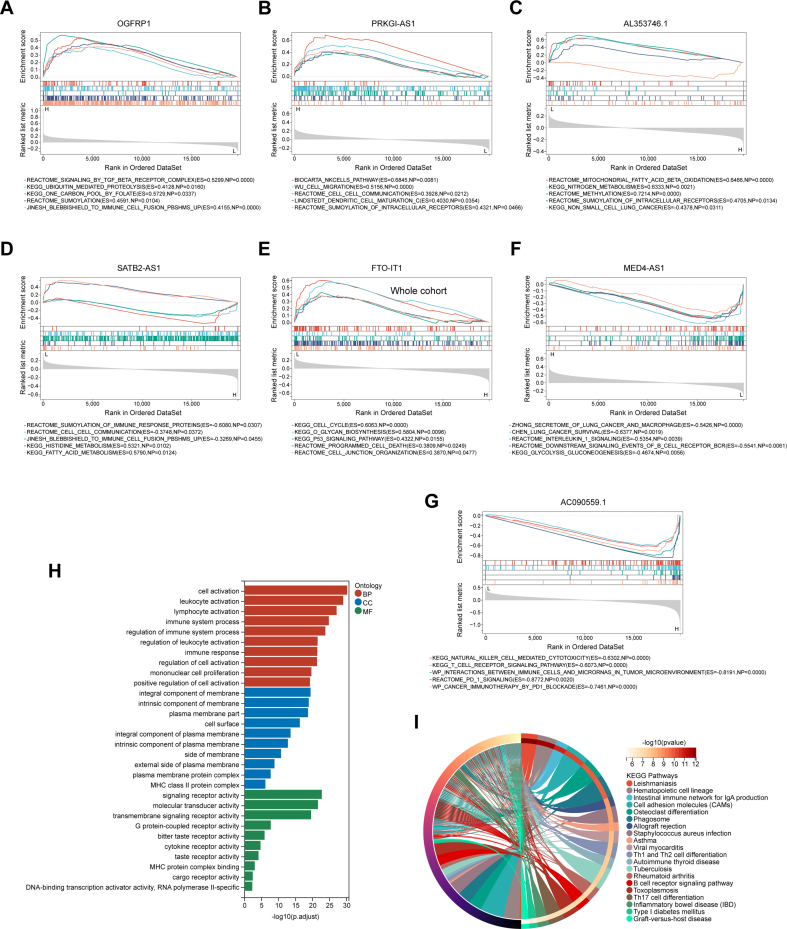
Functional analysis of the 7-SR-lncRNAs signature. **(A**–**G)** Gene Set Enrichment Analysis (GSEA) of OGFRP1 (A), PRKG1-AS1 (B), AL353746.1 (C), SATB2-AS1 (D), FTO-IT1 (E), MED4-AS1 (F), and AC090559.1 (G). The top 5 differentially regulated pathways were exhibited. **(H)** Gene Ontology (GO) enrichment analysis of 7-SR-lncRNAs-related mRNAs. **(I)** Kyoto Encyclopedia of Genes and Genomes (KEGG) pathway enrichment analysis of 7-SR-lncRNAs-related mRNAs. SR-lncRNAs, sumoylation-related lncRNAs.

**Figure S8:**
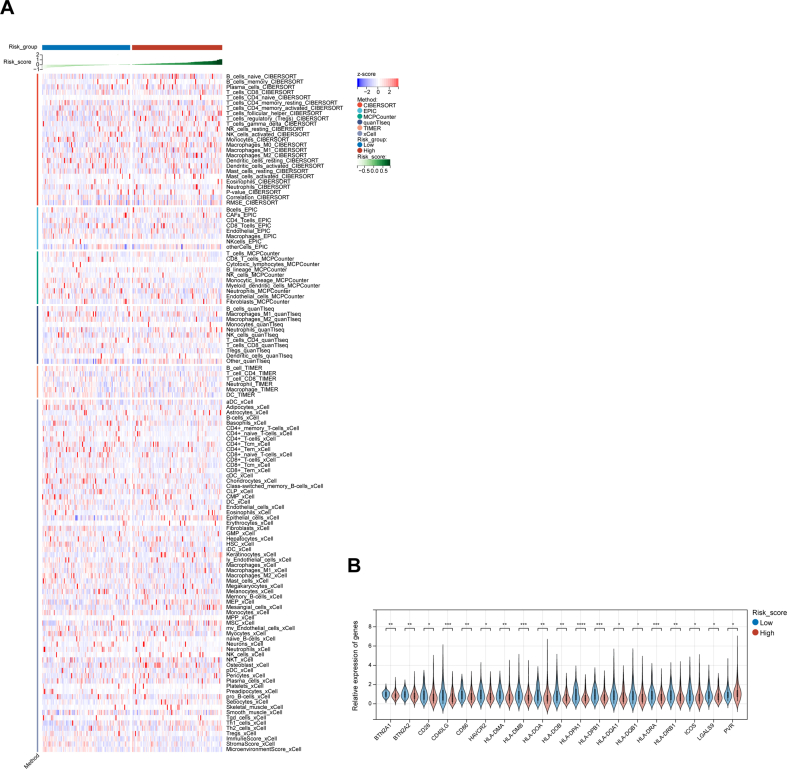
Differences in the immune landscape between low- and high-risk groups. **(A)** Infiltrated immune cell comparisons across low- and high-risk groups using CIBERSORT, EPIC, MCPCounter, quanTIseq, TIMER, and xCell algorithms. **(B)** The differentially expressed immune checkpoint genes between low- and high-groups.

**Figure S9:**
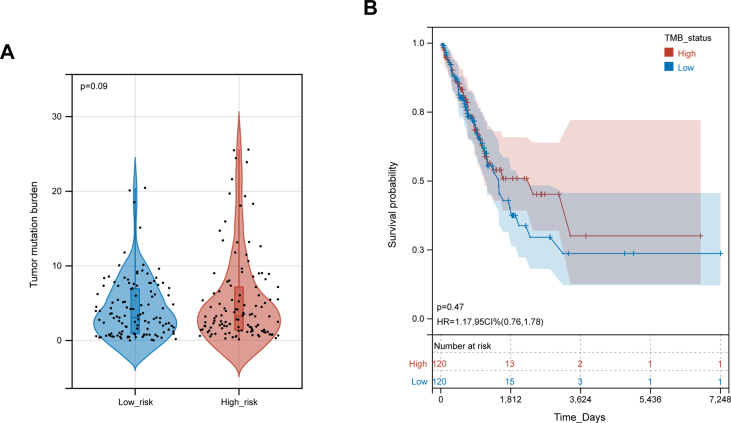
Landscape of mutation profiles in low- and high-risk lung adenocarcinoma patients. **(A)** The difference in tumor mutation burden (TMB) between low- and high-risk groups. **(B)** Kaplan–Meier curves for overall survival of the high-TMB and low-TMB groups.

**Figure S10:**
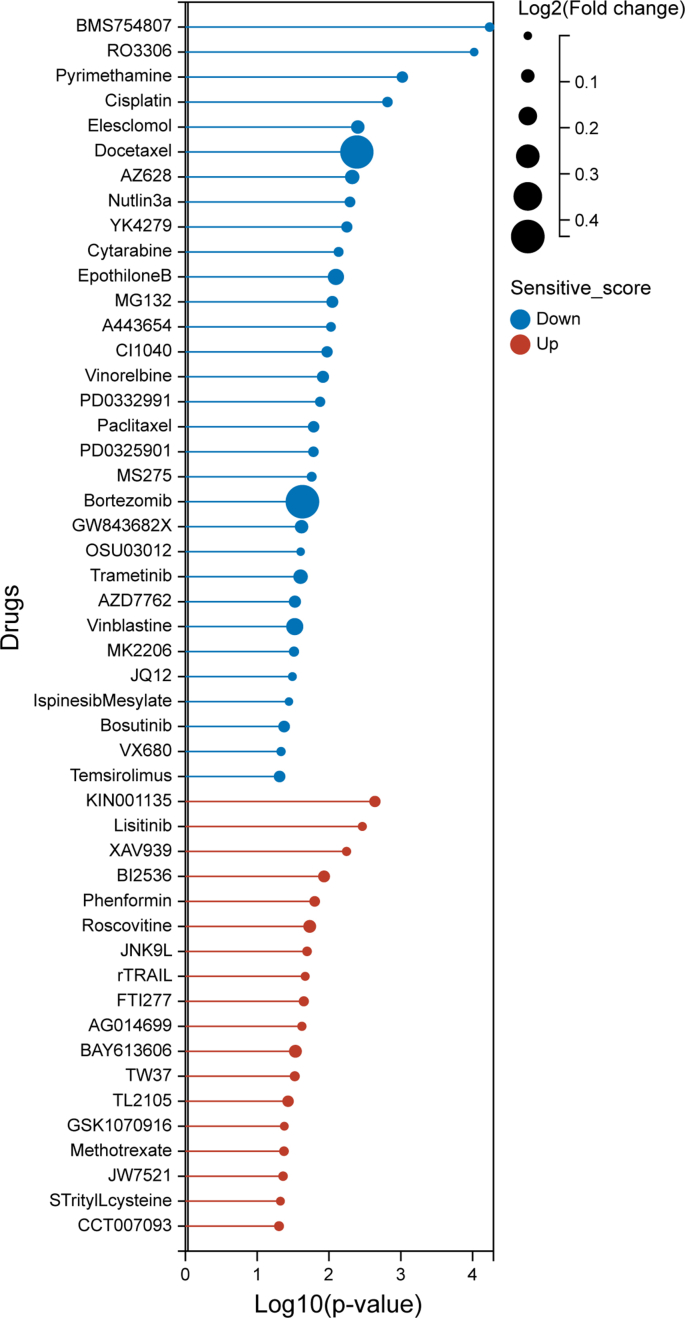
Differentially sensitive drugs between low- and high-risk groups analyzed with “pRRophetic” R package

**Figure S11:**
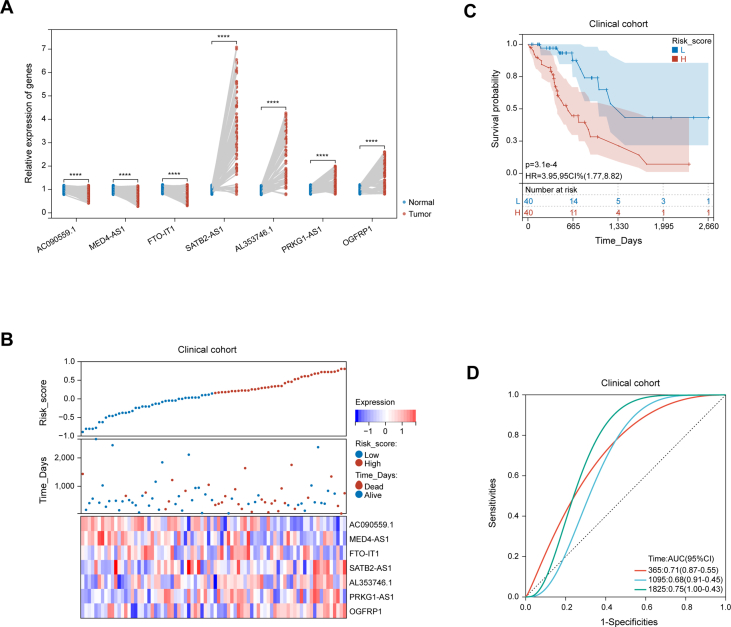
External validation of the 7-SR-lncRNAs signature in the clinical cohort. **(A)** The expression levels of 7 SR-lncRNAs in 80 paired samples of lung adenocarcinoma and adjacent normal tissues were examined by quantitative reverse transcription PCR. **(B)** The distribution of risk scores, OS status, and expression profiles of the 7 SR-lncRNAs in the clinical cohort. **(C)** Kaplan–Meier curve for OS in the clinical cohort. **(D)** The receiver operating characteristic (ROC) curves show the potential of the 7-SR-lncRNAs signature in predicting 1-, 3-, and 5-year OS in the clinical cohort. SR-lncRNAs, sumoylation-related lncRNAs; OS, overall survival.

**Figure S1:**
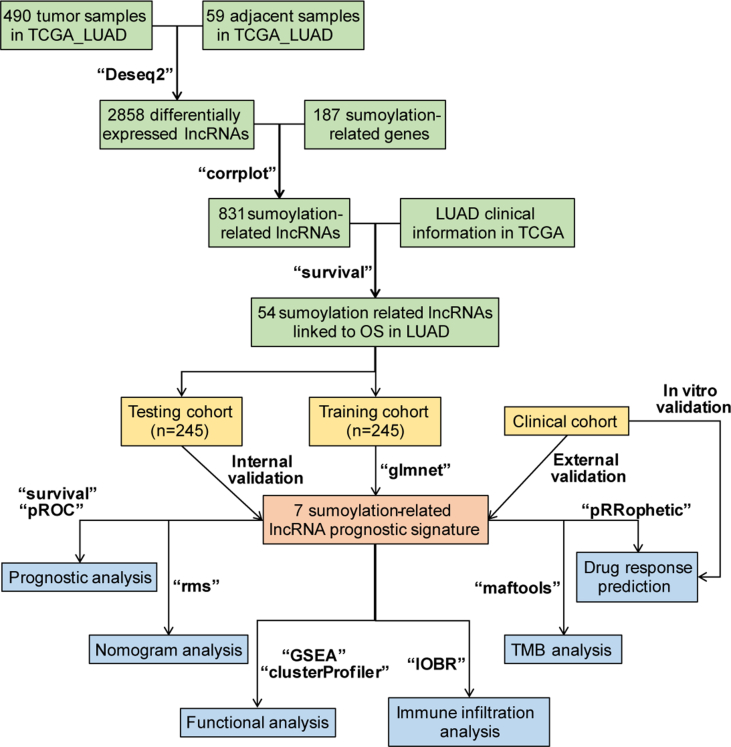
Flowchart of this study.

**Figure S2:**
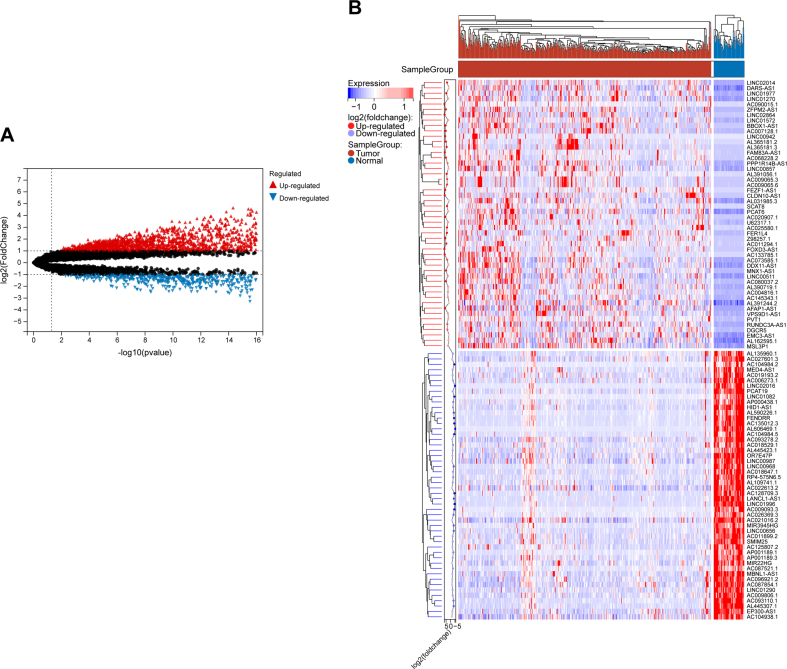
Identification of differentially expressed lncRNAs in lung adenocarcinoma. **(A)** The volcano plot exhibited 2003 up-regulated and 855 down-regulated differentially expressed lncRNAs (DE-lncRNAs). **(B)** The heatmap demonstrates the differential expression of the top 50 up-regulated and 50 down-regulated DE-lncRNAs in lung adenocarcinoma and normal tissues

**Figure S3:**
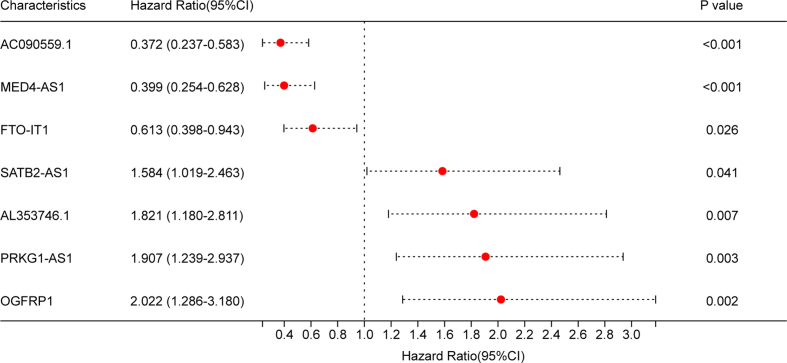
A 7-sumoylation-related-lncRNAs prognostic signature was finally created using stepwise multivariate Cox regression analysis.

**Figure S4:**
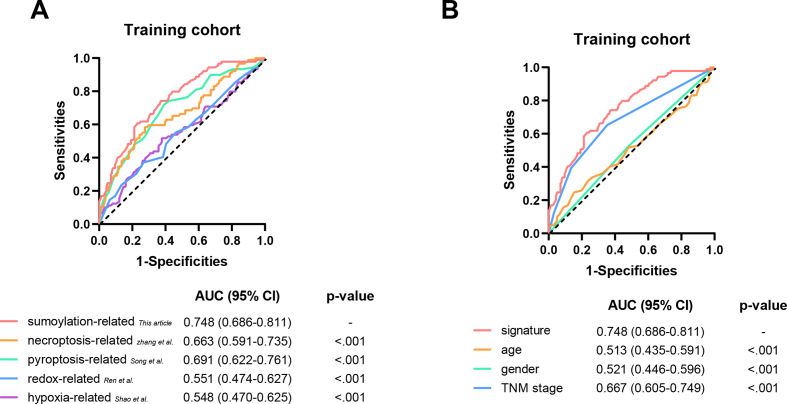
Comparison of prognostic predictive value between the 7-sumoylation-related-lncRNAs risk signature and previously published prognostic models **(A)** and clinicopathological risk factors **(B)**.

**Figure S5:**
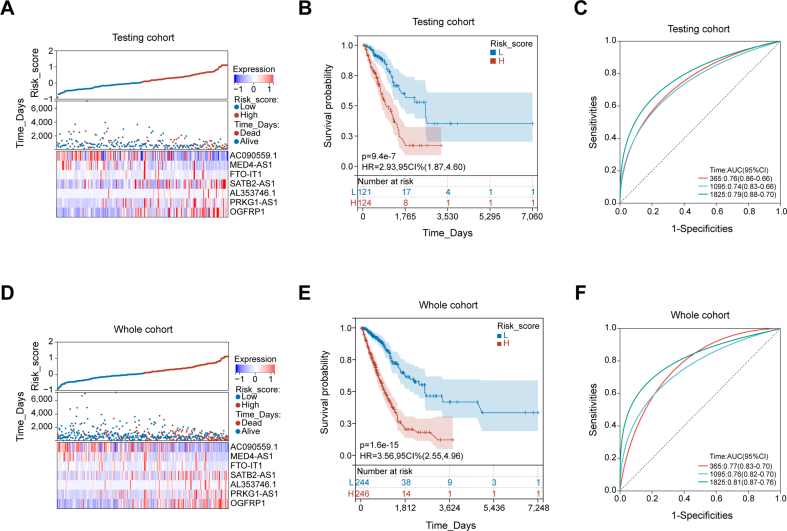
Prognostic value of the 7-SR-lncRNAs risk signature in the testing cohort and whole cohort. **(A)** The distribution of risk scores, OS status, and expression profiles of the 7 SR-lncRNAs in the testing cohort. **(B)** Kaplan–Meier curve for OS in the testing cohort. **(C)** The receiver operating characteristic (ROC) curves show the potential of the 7-SR-lncRNAs signature in predicting 1-, 3-, and 5-year OS in the testing cohort. **(D)** The distribution of risk scores, OS status, and expression profiles of the 7 SR-lncRNAs in the whole cohort. **(E)** Kaplan–Meier curve for OS in the whole cohort. **(F)** The ROC curves show the potential of the 7-SR-lncRNAs signature in predicting 1-, 3-, and 5-year OS in the whole cohort. SR-lncRNAs, sumoylation-related lncRNAs; OS, overall survival.
