# Supplementary material for: Construction and validation of a novel SUMOylation-related lncRNAs signature for predicting the prognosis, tumor immune microenvironment, and therapeutic sensitivity of lung adenocarcinoma
Source: Genes Dis. 2024 May 28;12(2):101338. doi: 10.1016/j.gendis.2024.101338 (PMC11742356; doi:10.1016/j.gendis.2024.101338)
Supplement: Multimedia component 1 [file mmc1.docx]

**Materials and methods**

**Data Processing**

The TCGA (https://cancergenome.nih.gov/) database was mined for information on LUAD, including RNA sequencing data and clinical details for 526 tumor samples and 59 normal samples[1]. Considering the probability of non-cancer deaths, patients with a survival time of ≤30 days and lacking expression data (n = 36) were excluded from the final cohort, leaving 490 LUAD patients. The Ensemble human genome browser GRCh38.p13 (http://asia.ensembl.org/index.html) was used for annotation and classification of protein-coding genes and lncRNAs.

**Identification of Prognostic Sumoylation-related LncRNAs**

Figure S1 is a flowchart depicting the analysis procedure. We utilized the R package "DESeq2" to find differentially expressed lncRNAs (DE-lncRNAs) with false discovery rate (FDR) < 0.01 and |log2 (fold change) | ≥ 1.0. From the Molecular Signatures Database (MSigDB, http://www.gseamsigdb.org/gsea/msigdb)[2], we obtained a list of 187 sumoylation-related genes (SRGs) (Table S1). The Pearson correlation coefficients were then used to quantify the relationship between the expression of SRGs and their related DE-lncRNAs. P < 0.001 and the absolute value of Pearson correlation coefficient more than 0.5 (|R| > 0.5) were employed as the stringent criterion for identifying sumoylation-related lncRNAs (SR-lncRNAs). Then, univariate Cox regression analysis for overall survival (OS) was employed to determine prognostic SR-lncRNAs with p < 0.01.

**Construction of the Sumoylation-related LncRNA Risk Signature**

Randomly and identically, the data from TCGA_LUAD were divided into two groups: a training cohort (n=245) and a testing cohort (n=245). Then, we used the "glmnet" R package to conduct a least absolute shrinkage and selection operator (LASSO) regression analysis[3], which screened variables and reduced their dimensionality to construct a more manageable and precise model in the training cohort. Least-squares methods typically produced the best values for lambda and related variables. Twenty SR-lncRNAs were found, with a minimum lambda of 0.050686024. Following this, multivariate Cox regression analysis was carried out, and 7 SR-lncRNAs were ultimately identified to create a more robust prognostic risk model.

**Evaluation and Validation**

Patients' risk scores were determined using regression coefficients and gene expression data. The median risk score was used to classify patients into low- and high-risk groups. Kaplan-Meier analysis and the long-rank test were utilized to see if there was a statistically significant difference in the OS rates between the two risk groups. R package "pROC" was used to examine ROC curves (AUCs for 1-, 3-, and 5- years OS) to evaluate the predictive model's sensitivity and specificity. The distribution of patients and the expression of biomarkers between low- and high-risk groups were visualized using risk score maps, survival status distribution maps, and expression heatmaps. The predictive power of this 7-SR-lncRNA signature was further verified in the testing and whole cohorts for internal validation, and in the clinical cohort for external validation. The clinical characteristics of LUAD patients in this study were summarized in Table S2.

**Nomogram Construction and Assessment**

To determine independent predictive markers, clinicopathological characteristics were analyzed using univariate and multivariate Cox regression analysis. Next, nomograms were built either including or excluding the 7-SR-lncRNA signature. By using nomograms, we estimated the prognosis of LUAD patients at 1, 3, and 5 years. The nomograms' predictive ability was calculated using the concordance index (C-index), and their precision and consistency were evaluated using calibration curves.

**Functional enrichment analysis**

Differences in enrichment scores between low- and high-risk groups were investigated using GSVA, with pathways from the Molecular Signatures Database (MSigDB) hallmark set serving as a reference. In addition, GSEA (version 4.1.0) was run on all 7 lncRNAs comprising the SR-lncRNAs signature. We used a cutoff of |Pearson correlation coefficient| >0.5 and P<0.01 to determine whether protein-coding genes were co-expressed with the 7 SR-lncRNAs. To decipher the roles of these correlated mRNAs, GO and KEGG functional enrichment analysis were performed. The analyses were mostly conducted utilizing the R packages "GSVA," "clusterProfiler," and "org.Hs.eg.db."

**Evaluation of the immune cell infiltration, immune function, and immune checkpoint genes**

Immune infiltration levels in LUAD patients were assessed using several well-established techniques[4], such as the cell-type identification by estimating relative subsets of RNA transcripts (CIBERSORT), estimating the proportions of immune and cancer cells (EPIC), microenvironment cell populationscounter (MCPCounter), quanTIseq, tumor immune estimation resource (TIMER), and xCELL. We also employed the Estimation of STromal and Immune cells in MAlignant Tumor tissues using Expression data (ESTIMATE) algorithm to compare immune and stromal activity in low- and high-risk groups[5]. Immune checkpoint genes (ICGs) were also analyzed for their differential expression between low- and high-risk patients.

**Somatic Variant Analysis**

On June 26, 2023, TCGA-LUAD gene somatic mutation data were retrieved from the Genomic Data Commons (GDC) database. The "maftools" R package was used to examine the downloaded Mutation Annotation Format (MAF) files of simple nucleotide variation (workflow type: varScan2 variant aggregation and masking)[6].

**Drug Sensitivity Prediction**

The "pRRophetic" R program has the potential to generate patient-specific medication sensitivities from gene expression data[7]. The sensitivity differences between low- and high-risk groups for 251 drugs from the "cpg2016" dataset in "pRRophytic" were evaluated. The Wilcoxon signed-rank test was utilized to compare the differences, and the top 10 findings are represented as violin plots.

**RNA Extraction and Quantitative Real Time-Polymerase Chain Reaction**

A total of 80 lung cancer samples and corresponding normal lung tissues were collected from patients who underwent surgical excision of LUAD at Shanghai Pulmonary Hospital. This study was approved by the Medical Ethics Committee of Shanghai Pulmonary Hospital. Written informed consent was obtained from each patient. The TRIzol reagent (Vazyme, Nanjing, China) was used to isolate total RNA, and the PrimeScript Reverse Transcriptase Reagent Kit (Takara Bio, Inc., Japan) was used to convert the RNA into complementary DNA (cDNA). In an ABI Step One Plus Real-Time PCR system (Applied Biosystems), we used TB Green® Premix Ex Taq^TM^ II (Takara, Tokyo, Japan) for amplification and detection. The endogenous control was β-actin. Sangon Biotech (Sangon, China) was responsible for synthesizing the primers. The sequences are shown in Table S3.

**Establishment of patient-derived LUAD cell lines**

Tumors were cut into pieces smaller than 1mm^3^ and then digested with Collagenase type IV (17104019, Thermo Fisher, MA, USA) at 1 mg/ml in DMEM/F12 (11320-033, Gibco, MA, USA) at 37°C for 3 hours to generate patient-derived LUAD cell lines (PDC). After that, three rounds of washing and resuspending in phosphate buffered saline (14190235, Thermo Fisher, MA, USA) were performed on the cells. The final cell suspensions were filtered through 70 µm cell strainers (352350, Falcon, CA, USA) and centrifuged at 600 g for 5 minutes at 4 °C. The cell pellets were re-suspended in DMEM (12430-054, Gibco, MA, USA), supplemented with 10% (v/v) fetal bovine serum (FBS) (10099, Gibco, MA, USA), and 1% penicillin-streptomycin (15070063, Gibco, MA, USA) and cultured.

**Dose-response IC50**

Approximately 5 × 10^3^ cells were planted per well into a 96-well plate 24 hours before drug treatment. Drugs were threefold diluted in DMSO and kept at 1% (v/v) across all drug concentrations and control. Triplicate samples were analyzed at each drug concentration. The viability of cells was assayed using CCK-8. A SpectraMax i3 microplate reader (Molecular Devices, CA, USA) was used to detect the luminescence signals at 450nm. Percentage cell viability was calculated by normalizing treatment wells' relative luminescence units to those of DMSO control wells. GraphPad Prism software was used to determine IC50 values.

**Statistical analysis**

For this study, we used R (version 4.1.2, R Foundation for Statistical Computing, Vienna, Austria) to conduct the statistical analysis. Pairwise comparisons across groups were conducted using the independent Student's t test for continuous data and the χ^2^ test for categorical data. Non-normally distributed variables were compared between the two groups using the Wilcoxon test. The log-rank test was used to compare two groups when Kaplan–Meier survival curves were generated. Statistical significance was defined as a p < 0.05 (*p < 0.05, **p < 0.01, ***p < 0.001).

**Reference**

1. Wang, Z., M.A. Jensen, and J.C. Zenklusen, *A Practical Guide to The Cancer Genome Atlas (TCGA).* Methods Mol Biol, 2016. **1418**: p. 111-41.

2. Liberzon, A., et al., *The Molecular Signatures Database (MSigDB) hallmark gene set collection.* Cell Syst, 2015. **1**(6): p. 417-425.

3. Liang, J.Y., et al., *A Novel Ferroptosis-related Gene Signature for Overall Survival Prediction in Patients with Hepatocellular Carcinoma.* Int J Biol Sci, 2020. **16**(13): p. 2430-2441.

4. Lu, H., et al., *Identifying a Novel Defined Pyroptosis-Associated Long Noncoding RNA Signature Contributes to Predicting Prognosis and Tumor Microenvironment of Bladder Cancer.* Front Immunol, 2022. **13**: p. 803355.

5. Yoshihara, K., et al., *Inferring tumour purity and stromal and immune cell admixture from expression data.* Nat Commun, 2013. **4**: p. 2612.

6. Mayakonda, A., et al., *Maftools: efficient and comprehensive analysis of somatic variants in cancer.* Genome Res, 2018. **28**(11): p. 1747-1756.

7. Geeleher, P., N. Cox, and R.S. Huang, *pRRophetic: an R package for prediction of clinical chemotherapeutic response from tumor gene expression levels.* PLoS One, 2014. **9**(9): p. e107468.
