# Supplementary material for: Construction and validation of a novel SUMOylation-related lncRNAs signature for predicting the prognosis, tumor immune microenvironment, and therapeutic sensitivity of lung adenocarcinoma
Source: Genes Dis. 2024 May 28;12(2):101338. doi: 10.1016/j.gendis.2024.101338 (PMC11742356; doi:10.1016/j.gendis.2024.101338)
Supplement: Multimedia component 3 [file mmc3.doc]

**Supplementary table 1. Clinical characteristics of LUAD patients in this study.**

| **Characteristics** | **TCGA_LUAD database** | | | **Clinical cohort**  **(n=80)** | **p-value** |
| --- | --- | --- | --- | --- | --- |
|  | **Training cohort**  **(n=245)** | **Testing cohort**  **(n=245)** | **Whole cohort**  **(n=490)** |  |  |
| **Gender**  Female  Male | 125  120 | 137  108 | 262  228 | 45  35 | .703 |
| **Age (years)**  <50  ≥50 | 18  227 | 20  225 | 38  452 | 8  72 | .891 |
| **TNM stage**  I  II | 132  57 | 132  64 | 264  121 | 44  21 | .938 |
| III | 40 | 40 | 80 | 13 |  |
| IV | 16 | 9 | 25 | 2 |  |
| **OS status**  Alive  Dead | 156  89 | 156  89 | 312  178 | 48  32 | .934 |
