# Supplementary material for: Construction and validation of a novel SUMOylation-related lncRNAs signature for predicting the prognosis, tumor immune microenvironment, and therapeutic sensitivity of lung adenocarcinoma
Source: Genes Dis. 2024 May 28;12(2):101338. doi: 10.1016/j.gendis.2024.101338 (PMC11742356; doi:10.1016/j.gendis.2024.101338)
Supplement: Multimedia component 4 [file mmc4.doc]

**Supplementary table 2. Primer pairs for qRT-PCR.**

| **Gene** | **Forward (5’-3’)** | **Reverse (5’-3’)** |
| --- | --- | --- |
| OGFRP1 | CGACCAGCAGGTCACTGATT | GCTCACTTCGCACAAGTCAC |
| PRKG1-AS1 | GCGAGCGTTATAGCAGACGA | TTGCAGAGCCTATCACCCCT |
| AL353746.1 | AGCAGCCTCTTTGCATGTAT | ATGGTGTCTGAGGGAAGTGA |
| SATB2-AS1 | AGAACCGGCGTTTCAGATGT | GAGGGATCAGCGAGCTACC |
| FTO-IT1 | GGCTTTGAAATCCAAATGGGATGA | TCAGCAGAAGGTCTTTAATCTAGT |
| MED4-AS1 | TCGGCTTAACATTACCTGGCA | CGGAGACCCTACCCAACTGA |
| AC090559.1 | GGTGATGGCTGCGTTTAAGT | AAAACAGCCCAGTTCCTCTG |
| β-actin | GGGAAATCGTGCGTGACATTAAG | TGTGTTGGCGTACAGGTCTTTG |
